# Spontaneous Rupture of the Imperforate Hymen in an Adolescent Girl with Hematocolpometra

**DOI:** 10.5402/2011/520304

**Published:** 2010-09-29

**Authors:** Zehra Kurdoglu, Mertihan Kurdoglu, Zehra Kucukaydin

**Affiliations:** Department of Obstetrics and Gynecology, Yuzuncu Yil University School of Medicine, 65300 Van, Turkey

## Abstract

*Background*. Imperforate hymen is usually treated with hymenotomy, and the management after its spontaneous rupture is not very well known. *Case*. In this paper, we present spontaneous rupture of the imperforate hymen in a 13-year-old adolescent girl with hematocolpometra just before a planned hymenotomy operation. The patient was managed conservatively with a satisfactory outcome. *Conclusion*. Hymenotomy may not be needed in cases with spontaneous rupture of the imperforate hymen if adequate opening for menstrual discharge is warranted.

## 1. Introduction

Imperforate hymen is reported at an approximate rate of 0,1% and occurs due to the incomplete canalization of the Mullerian system and the urogenital system [1]. The treatment of this condition that can be easily diagnosed through gynecological examination is surgical [2]. 

In this paper, we present an adolescent girl with a spontaneous ruptured imperforate hymen who is the first case in the literature.

## 2. Case Report

**Figure 1:**
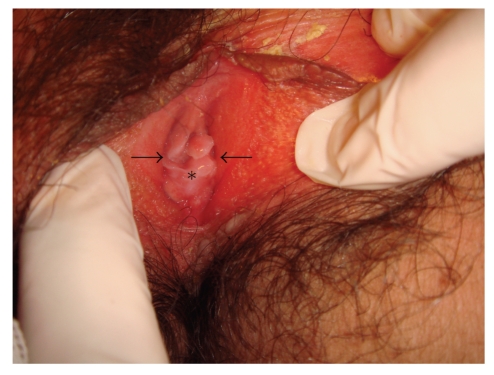
The appearance of the hymen two months after spontaneous rupture, with deflorations at the 3 and 9 o'clock positions (arrows) and the lower half closed (asterisk).

Gynecological examination of a 13-year-old female patient admitted to our clinic with a cyclic abdominal pain and amenorrhea revealed bulging out of a thinned hymen from the introitus with a blue reflection. Furthermore, on rectal examination, a mass lesion of approximately 10 cm in size was palpated and ultrasonography showed a 14 × 8 cm mass lesion compatible with hematocolpometra. The patient was hospitalized with a diagnosis of imperforate hymen, and hymenotomy was planned. She had severe abdominal and pelvic pain with a dark bloody discharge on the night just before the operation. Reexamination revealed an opening with irregular borders in the upper half of the hymenal ring. The ruptured area was assessed to be adequate to provide menstrual drainage, and the patient was decided to be followedup. She was also informed about the possibility of reclosure of the hymen. On her next visit two months later, she described a normal menstrual period. Although lower half of the hymen was still closed, it was open in upper half with deflorations at 3 and 9 o'clock positions (Figure 1). Hematocolpometra did not exist any more. In her last visit, one year later, her menstruations were still regular and adequate without any complaint. 

## 3. Discussion

Cyclical pelvic pain 2.5 to 4 years after thelarche is the usual presentation in a teenager with imperforate hymen [3]. Sometimes it can be fairly asymptomatic, and rarely there is obstruction of the urinary tract from the hematocolpos [4]. With the improving effect of estrogenization on healing, hymenal resection is best performed in adolescence [3].

This case report is an example of spontaneous hymenal rupture and no similar case report was found in the literature. Bakos and Berglund [5] reported a case of acute abdomen due to bilateral hematosalpinx and unilateral rupture of the hematosalpinx owing to an imperforate hymen. In that report, rupture of the tuba was thought to be as a result of distention due to the increased pressure along with hematometrocolpos. Similarly, in our case, we think that the retrograde accumulation of menstrual blood leaded to hematometrocolpos, increased intravaginal pressure and perforated the hymen. Therefore, when there is an increase in the intensity of pain of an adolescent girl with hematocolpometra, spontaneous rupture of the imperforate hymen should be kept in mind and examination should be repeated.

It may be interesting to see what will happen when this girl gets married. There is a possibility that she can develop a rigid hymen that has to be surgically removed.

As a conclusion, the time of spontaneous rupture cannot be predicted, and, therefore, waiting for that in an adolescent girl with imperforate hymen should not be recommended. However, hymenotomy may not be needed in cases with spontaneous rupture of imperforate hymen if adequate opening for menstrual discharge is warranted. Close followup and operating only the ones with reclosure or inadequate hymenal opening may be logical as far as a minimal invasive approach is concerned.
